# Theanine, a Tea-Leaf-Specific Amino Acid, Alleviates Stress through Modulation of Npas4 Expression in Group-Housed Older Mice

**DOI:** 10.3390/ijms24043983

**Published:** 2023-02-16

**Authors:** Keiko Unno, Kyoko Taguchi, Tomokazu Konishi, Makoto Ozeki, Yoriyuki Nakamura

**Affiliations:** 1 Tea Science Center, University of Shizuoka, 52-1 Yada, Suruga-ku, Shizuoka 422-8526, Japan; 2 Faculty of Bioresources Sciences, Akita Prefectural University, Shimoshinjo Nakano, Akita 010-0195, Japan; 3 Taiyo Kagaku Co., Ltd., 1-3 Takaramachi, Yokkaichi 510-0844, Japan

**Keywords:** DNA methyltransferase, glucocorticoid receptor, group housing, IL-1β, Npas4, REST, stress, theanine

## Abstract

Group rearing is a common housing condition, but group-housed older mice show increased adrenal hypertrophy, a marker of stress. However, the ingestion of theanine, an amino acid unique to tea leaves, suppressed stress. We aimed to elucidate the mechanism of theanine’s stress-reducing effects using group-reared older mice. The expression of repressor element 1 silencing transcription factor (REST), which represses excitability-related genes, was increased in the hippocampus of group-reared older mice, whereas the expression of neuronal PAS domain protein 4 (Npas4), which is involved in the regulation of excitation and inhibition in the brain, was lower in the hippocampus of older group-reared mice than in same-aged two-to-a-house mice. That is, the expression patterns of REST and Npas4 were found to be just inversely correlated. On the other hand, the expression levels of the glucocorticoid receptor and DNA methyltransferase, which suppress Npas4 transcription, were higher in the older group-housed mice. In mice fed theanine, the stress response was reduced and Npas4 expression tended to be increased. These results suggest that Npas4 expression was suppressed by the increased expression of REST and Npas4 downregulators in the group-fed older mice, but that theanine avoids the decrease in Npas4 expression by suppressing the expression of Npas4 transcriptional repressors.

## 1. Introduction

L-theanine (γ-glutamylethylamine) is a non-protein amino acid that is rarely found in plants other than tea (*Camellia sinensis*) and is the major amino acid in Japanese green tea, which is a widely consumed beverage associated with human health [1]. Since theanine is structurally similar to glutamic acid and is taken into the brain through the blood–brain barrier [2], its function in the brain has been studied. For example, it has been reported that theanine has a relaxing effect, as alpha waves have been observed to significantly increase in the brain after its ingestion [3]. In addition, animal experiments and clinical studies in humans have shown that theanine offers excellent stress-relieving effects [4,5,6,7,8,9]. Theanine acts via glutamate receptors but binds rather tightly to glutamine receptors [10]. Therefore, it has been proposed that theanine modulates the glutamate–glutamine cycle and inhibits the release of excess excitatory neurotransmitter glutamate [10]. In addition, neurogenesis in the hippocampus is an important target in stress-induced diseases [11], and theanine has been reported to upregulate the expression of Slc38a1, one of the glutamine transporter isoforms, and promote neuronal differentiation and proliferation [12].

On the other hand, neuronal Per-Arnt-Sim (PAS) domain protein 4 (Npas4) is a recently discovered calcium-dependent transcription factor that regulates the activation of genes involved in the homeostatic regulation of the excitatory–inhibitory balance within neural circuits [13,14]. Npas4 expression is reported to decrease under various stress conditions in mice and rats [14]. In addition, it has been shown that rats with higher expression of Npas4 in the hippocampus due to stress recover more quickly from stress than those with lower expression [15]. Higher expression of Npas4 may be important for stress tolerance.

When the effects of territorial confrontation stress were examined in male mice, adrenal hypertrophy was observed after 24 h and continued for more than a week thereafter [8]. This indicates that the hypothalamus–pituitary–adrenal (HPA) axis was activated by the stress the mice were experiencing. Although adrenal hypertrophy has been observed in all strains of male mice examined to date and is a reliable marker of stress, theanine intake has been shown to suppress such stress-induced adrenal hypertrophy [8,16].

We have also found that different strains of mice differ in their sensitivity to stress. For example, SAMP10, an accelerated aging model mouse, is vulnerable to stress, whereas ddY, an outbred mouse that is widely used in Japan, is resistant to stress [17,18]. In SAMP10, confrontational stress caused early brain atrophy, which accelerated with aging, whereas in ddY, the stress load caused brain atrophy, but the atrophy subsequently recovered [17]. In addition, SAMP10 showed a shortening of the life span due to stress loading [7], while ddY showed no change in life span (unpublished data). These results suggest that ddY is a stress-tolerant strain. However, ddY mice developed brain atrophy during group rearing, which was suppressed by theanine intake, suggesting that long-term group rearing is stressful for ddY mice [17].

Based on these studies, we attempted to elucidate why ddY mice, which show stress tolerance under confrontational stress conditions, become more stressed with age, even under relatively low-stress group housing conditions. In addition, we attempted to elucidate the targets of theanine’s stress-relieving effects.

## 2. Results

### 2.1. Body, Adrenal Glands, Thymus, and Cerebrum Weights

**Figure 1:**
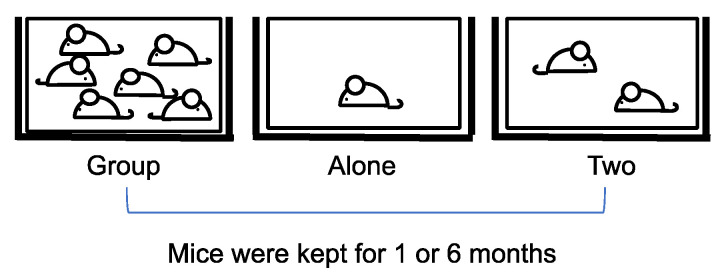
Housing conditions: group, single, and double.

Four-week-old ddY mice were kept for one or six months in one of three different rearing conditions: group housing of six mice per cage, single housing of one mouse per cage, and double housing of two mice per cage (Figure 1). For each housing condition, the mice were given theanine, an amino acid unique to tea leaves that has been shown to reduce stress, in free drinking water, and were compared to those in the same housing condition with normal water intake. During dissection, the body, adrenal gland, thymus, and cerebrum weights were measured.

**Figure 2:**
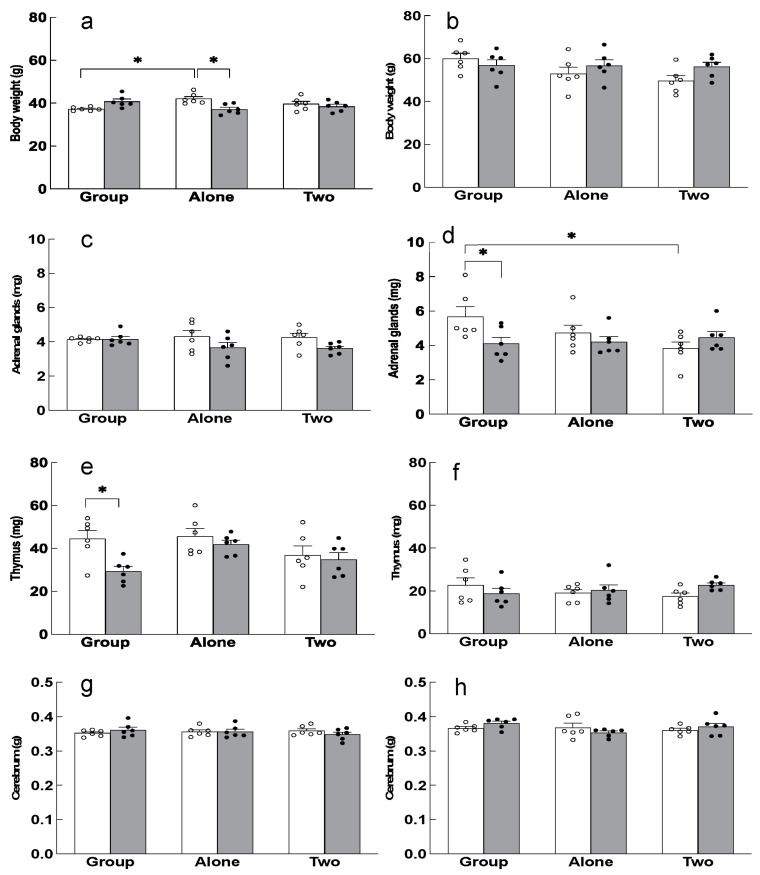
Body, adrenal glands, thymus, and cerebrum weights in the group-, single-, and double-housed mice. Body weight of mice in each rearing condition for 1 month (2 months old) (**a**) and 6 months (7 months old) (**b**). Weight of mice’s adrenal glands in each rearing condition for 1 month (2 months old) (**c**) and 6 months (7 months old) (**d**). Thymus weight of mice in each rearing condition for 1 month (2 months old) (**e**) and 6 months (7 months old) (**f**). Cerebrum weight of mice in each rearing condition for 1 month (2 months old) (**g**) and 6 months (7 months old) (**h**). Each column bar represents the mean ± SEM (n = 6) overlaid on scatter plots (* *p* < 0.05, Tukey’s honestly significant difference method). Open columns and white dots are control mice. Closed columns and black dots are theanine-ingesting mice.

In mice reared alone for one month, significant weight gain was observed, which was suppressed by theanine intake (Figure 2a). Mice reared for six months (seven months old) weighed 1.5 times more than those reared for one month (two months old), but there was no effect of rearing conditions (Figure 2b). No differences in the weight of adrenal glands were observed between rearing conditions (Figure 2c). However, in seven-month-old mice, the adrenal glands were significantly enlarged in the group-housed mice compared to the two-to-a-house mice (Figure 2d). Theanine intake significantly suppressed adrenal hypertrophy in the group-housed older mice. This indicated that group housing increased stress in the mice as they aged. Atrophy of the thymus gland was observed in the theanine-fed group-housed mice after one month (Figure 2e). The wet weight of the thymus did not differ in the six-month rearing condition but was considerably lower than the wet weights in the one-month rearing condition (Figure 2f). The wet weight of the cerebrum was not affected by the housing conditions, duration of rearing, or theanine intake (Figure 2g,h).

### 2.2. Stress-Related Gene Expression in the Hippocampus and Cerebral Cortex

**Figure 3:**
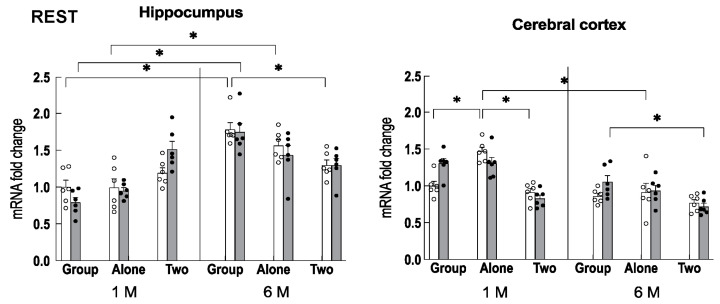
The expression of REST in the hippocampus and cerebral cortex under each rearing condition for 1 month (1 M, 2 months old) and 6 months (6 M, 7 months old) in control mice (open column) and theanine-ingesting mice (closed column). Each column bar represents the mean ± SEM (n = 6) overlaid on scatter plots (* *p* < 0.05, Tukey’s honestly significant difference method).

Since the transcription factor REST (repressor element 1 silencing transcription factor) plays an important role in inhibiting neuronal excitation during aging [19], we examined the expression of REST in the hippocampus and cerebral cortex. The results showed that the expression of REST was increased in the hippocampus of older mice that had been housed in a group compared to the two-to-a-house mice of the same age (Figure 3). The difference was similar to the difference in the adrenal weight of control group-reared older mice. No effect of theanine on the expression of REST was observed. In the cerebral cortex, its expression was high in young mice housed alone but was suppressed in the older mice. The expression pattern differed between the hippocampus and cortex.

**Figure 4:**
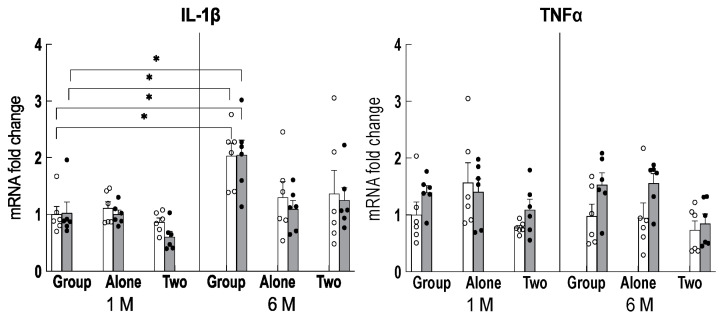
Changes in the pro-inflammatory gene expression in the hippocampus of mice reared for 1 month (1 M, 2 months old) and 6 months (6 M, 7 months old) in each housing condition for the control mice (open column) and the theanine-ingesting mice (closed column). Each column bar represents the mean ± SEM (n = 6) overlaid on scatter plots (* *p* < 0.05, Tukey’s honestly significant difference method).

Next, since neuroinflammation increases with age [20], we examined changes in the expression of genes involved in inflammation, such as *IL-1β* and *TNFα*, in the hippocampus. The results showed that the expression of *IL-1β* was increased in the hippocampus of group-housed older mice compared to the two-to-a-house mice (Figure 4), and theanine intake did not affect *IL-1β* expression.

### 2.3. Gene Expression Changes in the Hippocampus of Group-Housed Mice at Two Months of Age

To examine the targets of theanine’s stress reduction effects in group housing, a comprehensive comparison of the genes altered by theanine intake in the hippocampus of mice that were group housed for one month was conducted. Since it was necessary to make a comparison with the older group, the basic younger group was examined. The main functions whose expressions were significantly decreased or increased by theanine intake are listed in Table 1. Among the genes belonging to the same function, more had their expression decreased by theanine intake rather than increased. In particular, there were 2.5 times more repressed genes in the category of “regulation of transcription, DNA-templated” than promoted genes. No biological data were available for genes belonging to “biological process”. A trend towards more downregulation than upregulation was also observed in other functions.

Next, the main genes whose expression was significantly decreased or increased by theanine intake are listed in Table 2. The transthyretin was thought to reflect the difference in the choroid plexus, which was slightly contaminated when the hippocampus was sampled [21]. Kcnj13 (potassium inwardly rectifying channel, subfamily J, member 13) has been reported to be involved in the regulation of cell excitability in the hippocampus via potassium transport [22]. Npas4 (neuronal PSA domain protein 4) is an important target for regulating responses to stress and promotes the development of inhibitory GABA synapses in excitatory pyramidal cells of the hippocampus. It also functions as a transcriptional enhancer [15]. Fos (FBJ osteosarcoma oncogene), Arc (activity-regulated cytoskeletal-associated protein), and Egr2 (early growth response 2) are all immediate early genes (IEGs) and are used as markers of neural activity, including stress responses [23]. It has been reported that DUSP1 (dual specificity phosphatase 1) is upregulated in the hippocampus during stress and causes depressive behavior [24]. Nr4a1 (clear receptor subfamily 4, group A, member 1) is also commonly used as a marker of stress [25]. On the other hand, the hemoglobin genes Hbb-b2 (hemoglobin, beta adult minor chain) and Hbb-a2 (hemoglobin alpha, adult chain 2) are not altered in acute stress, but their expression has been reported to increase during chronic social stress [26]. Txnip (thioredoxin-interacting protein) increases in the hippocampus with chronic stress [27].

### 2.4. The Effect of Age on Npas4 Gene Expression in the Hippocampus and Cerebral Cortex

**Figure 5:**
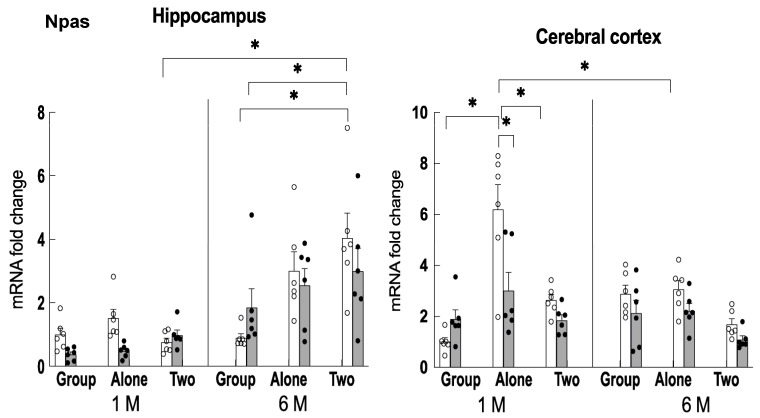
Changes in Npas4 gene expression in the hippocampus and cerebral cortex of mice reared for 1 month (1 M, 2 months old) and 6 months (6 M, 7 months old) in each housing condition. Open column: control mice. Closed column: theanine-ingesting mice. Each column bar represents the mean ± SEM (n = 6) overlaid on scatter plots (* *p* < 0.05, Tukey’s honestly significant difference method).

We examined how the expression levels of genes that showed significant changes in expression in Section 2.3 would subsequently change as the mice continued group housing until they reached the age of seven months. We first focused on *Npas4*, which has been found to be one of the important targets of theanine [17], and examined changes in its expression in the hippocampus and cerebral cortex (Figure 5). In the hippocampus, *Npas4* expression was high in older mice raised in two-to-a-house. Thus, it was shown that increased *Npas4* expression was present in older mice even under low-stress conditions. In group-reared older mice, however, *Npas4* expression was not increased. The changes in *Npas4* expression in the hippocampus of older mice were inversely correlated with adrenal hypertrophy and REST expression levels.

On the other hand, in the cerebral cortex, a significant increase in *Npas4* expression was observed in the younger mice (two months of age) reared alone, but the increase was suppressed by theanine ingestion as well as in older mice. *Npas4* showed differences in expression in the hippocampus and cerebral cortex in different rearing conditions.

### 2.5. The Effect of Age on IEG Expression in the Hippocampus

**Figure 6:**
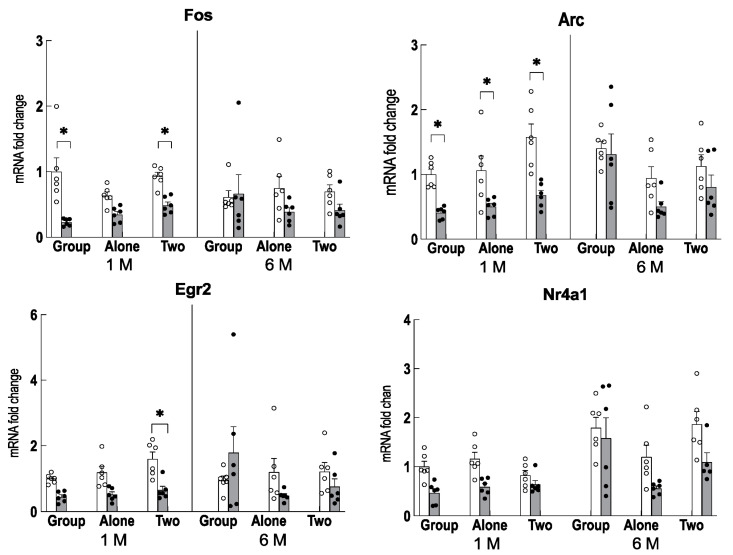
Changes in IEG expression in the hippocampus and cerebral cortex of mice reared for 1 month (1 M, 2 months old) and 6 months (6 M, 7 months old) in each housing condition. Open column: control mice. Closed column: theanine-ingesting mice. Each column bar represents the mean ± SEM (n = 6) overlaid on scatter plots (* *p* < 0.05, Fisher’s exact probability test).

In the hippocampus of younger mice, the expression of IEGs such as *Fos, Arc*, and *Egr2* was suppressed by theanine intake in young mice, but no change in expression was observed due to aging or rearing conditions (Figure 6). The expression of *Nr4a1* increased with aging, but no significant changes were observed with rearing conditions (Figure 6).

### 2.6. Expression of Glucocorticoid Receptor and DNA Methyltransferase, which Downregulate Npas4

**Figure 7:**
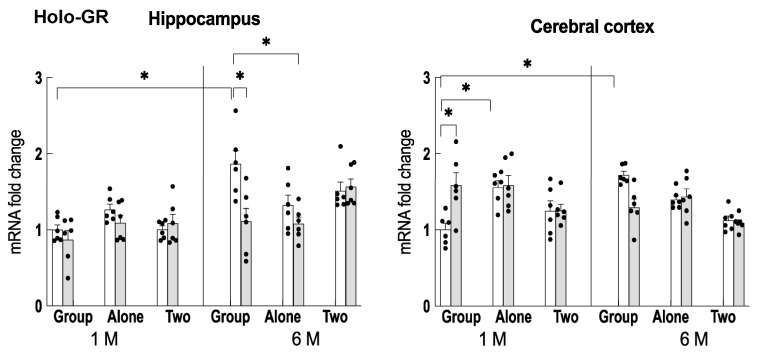
Changes in holo-GR gene expression in the hippocampus and cerebral cortex of mice reared for 1 month (1 M, 2 months old) and 6 months (6 M, 7 months old) in each housing condition. Open column: control mice. Closed column: theanine-ingesting mice. Each column bar represents the mean ± SEM (n = 6) overlaid on scatter plots (* *p* < 0.05, Tukey’s honestly significant difference method).

**Figure 8:**
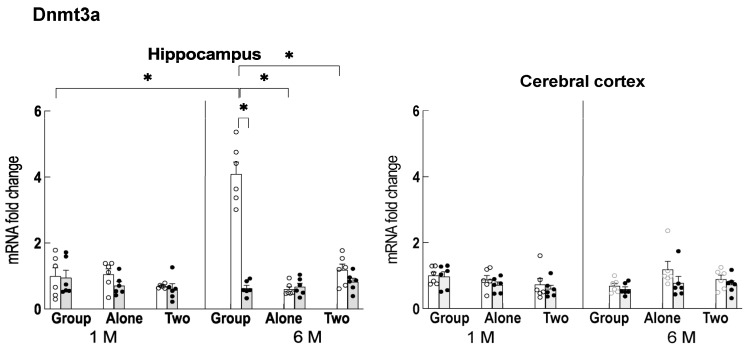
Changes in Dnmt3a gene expression in the hippocampus and cerebral cortex of mice reared for 1 month (1 M, 2 months old) and 6 months (6 M, 7 months old) in each housing condition. Open column: control mice. Closed column: theanine-ingesting mice. Each column bar represents the mean ± SEM (n = 6) overlaid on scatter plots (* *p* < 0.05, Tukey’s honestly significant difference method).

The transcription of Npas4 has been reported to be downregulated via the binding of agonist-bound glucocorticoid receptor (holo-GR) and DNA methylation [28,29]. Therefore, the expression of holo-GR was examined in the hippocampus and cerebral cortex. The results showed that its expression in the hippocampus was significantly higher in group-reared older mice, and its expression was reduced by theanine intake (Figure 7). In the cerebral cortex, on the other hand, the expression was upregulated in younger single-housed mice and older group-fed mice. Theanine ingestion also increased the expression of holo-GR in older group-reared mice. Among the DNA methyltransferases, Dnmt3a was significantly upregulated in the hippocampus of older group-fed mice and was suppressed by theanine ingestion (Figure 8).

## 3. Discussion

**Figure 9:**
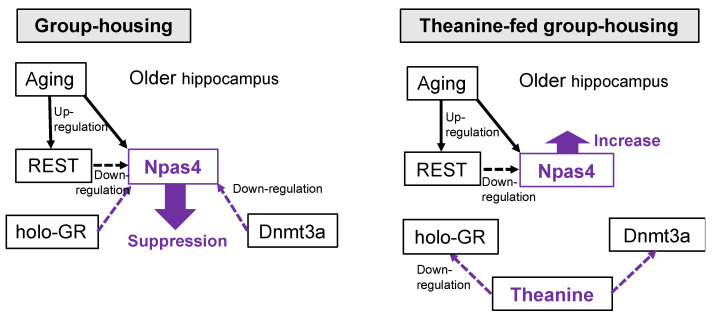
Factors relating to changes in Npas4 expression due to stress and aging, and the role of theanine. Solid line: upregulation. Dashed line: downregulation.

In control older mice, the degree of adrenal hypertrophy was highest in the group-reared condition, followed by the solo-reared condition, and was lowest in the two-to-a-house reared condition. This order is similar to the expression pattern of REST, whereas Npas4 expression was inversely correlated. REST is closely associated with glutamatergic innervation and is involved in maintaining the balance between neuronal excitation and inhibition [30]. Npas4 is an important target for regulating the response to stressors, and high expression of Npas4 has been reported to be advantageous for stress management [15]. As neural excitability increases with aging [19], suppression of excitability is particularly important in stress-loaded aging mice. We found that an increase in Npas4 expression occurred with aging (Figure 6). However, REST has been reported to suppress Npas4 expression [31]. Thus, we further considered the cause of the suppression of Npas4 expression in the group-fed older mice. We found that holo-GR and Dnmt3a were elevated in the group-reared older mice. Transient stress has been reported to suppress Npas4 expression in the brain by the binding of holo-GR to its promoter [28], and the long-term stress load causes the decreased expression via the DNA methylation of its promoter portion [29]. That is, the upregulation of holo-GR and Dnmt3a may be involved in the decreased Npas4 expression in the hippocampus of the group-housed older mice. Therefore, it is possible that Npas4 expression was suppressed due to these relationships (Figure 9).

On the other hand, adrenal hypertrophy was significantly suppressed in aged mice fed theanine compared to controls, even under group-rearing conditions. Although theanine intake did not affect the expression of REST, the expression of holo-GR and Dnmt3a was significantly suppressed. Therefore, these relationships suggest that the repression of Npas4 expression was reversed to some extent, resulting in stress reduction (Figure 9). Although the increase in Npas4 expression relative to controls was not statistically significant, the subtle tuning in the regulation of neuronal excitatory/inhibitory balance may be significant.

Since mice are social animals, group housing is regarded as a relatively low-stress rearing condition; as long as no hurtful aggression is observed in the group, they are considered to be kept without problems. Group housing methods are widely recommended when breeding mice for experiments. The ddY mice used in this experiment grew fast, resulting in a 1.5-fold increase in body weight at seven months compared to two months. Therefore, the stress of overcrowding may be a factor, but the effects of aging are likely to be important in group-reared older mice.

In the solo housing condition, Npas4 expression was increased in the cerebral cortex of younger mice, and no significant adrenal hypertrophy was observed in older mice. This may suggest that increased expression of Npas4 during stress loading is necessary for the acquisition of tolerance to stress.

Acute elevation of glucocorticoids suppresses cytokine production in the brain, but central catecholamines stimulate the release of IL-1β from microglia [32], which is thought to increase neuroinflammation in the brain due to stress. It has also been reported that chronic stress promotes the release of pro-inflammatory cytokines [33], but theanine was suggested not to be involved in the cytokine-mediated stress response. Decreased Npas4 expression may increase inflammatory factors [34], but IL-1β may not affect Npas4 expression.

In this study, we were able to elucidate part of the regulatory mechanism of Npas4 expression by theanine in older mice under stressful conditions, but further studies are needed to elucidate the molecular mechanism of theanine’s stress-reducing effect. It is also necessary to clarify how the strong binding of theanine to glutamine receptors, which has been found so far, acts on the regulation of Npas4 expression.

## 4. Materials and Methods

### 4.1. Animals

Four-week-old male ddY mice were purchased from Japan SLC Co. Ltd. (Shizuoka, Japan) and kept in conventional conditions in a temperature- and humidity-controlled room with a 12–12 h light–dark cycle (light period, 08:00–20:00; temperature, 23 ± 1 °C; relative humidity, 55 ± 5%). Mice were fed a normal diet (CE-2; Clea Co. Ltd., Tokyo, Japan) and water ad libitum. All experimental protocols were approved by the University of Shizuoka Laboratory Animal Care Advisory Committee (approval no. 195241) and were in accordance with the guidelines of the US National Institutes of Health for the care and use of laboratory animals.

### 4.2. Experimental Design

For the experiment, 72 mice were prepared and divided into 12 groups. Group-housed mice were housed with six mice per cage (Figure 1). Mice in single housing were housed with one per cage. Mice in two-to-a-house were housed with two to a cage. Six groups of 36 mice each consumed theanine (Taiyo Kagaku Co. Ltd., Yokkaichi, Japan) in water at a concentration of 20 µg/mL from one month of age. These mice drank about 10 mL of water daily. Another six groups of 36 mice (control) consumed water (Table 3).

Theanine doses are based on previous experimental results [16]. Theanine at 5–100 μg/mL has been found to similarly inhibit adrenal hypertrophy, with 20 μg/mL used in previous experiments.

### 4.3. Measurement of DNA Microarray and Principal Component Analysis

The mice housed in groups of 6 for 1 month were fed water containing theanine or nothing (control). The hippocampus was removed from each mouse and frozen immediately. Total RNA was obtained from the hippocampus using a purification kit (NucleoSpin^®^ RNA, 740955, TaKaRa Bio Inc., Shiga, Japan). Biotinylated cRNA was synthesized from this total RNA using One-Cycle Target Labeling and Control Reagents (Affymetrix, Santa Clara, CA, USA) and hybridized to Total RNA Mouse Gene 1.0 ST Array (Affymetrix). Three biological replicates were performed for each group. The raw data were normalized using the SuperNORM data service (Skylight Biotech Inc., Akita, Japan) [35]. The significance of theanine ingestion was tested by two-way ANOVA at *p* < 0.001 [36]. To compare the effects of theanine intake, principal component analysis (PCA) was performed on ANOVA-positive genes [37,38].

### 4.4. Quantitative Real-Time Reverse Transcription PCR (qRT-PCR)

Mice at 2 and 7 months of age who fed water containing theanine (~5 mg/kg) or not were used for this analysis. Isoflurane was used to anesthetize those mice. The hippocampi and prefrontal cortex removed from the brain of each mouse were immediately frozen. Total RNA was isolated from homogenized brain samples using a purification kit (NucleoSpin^®^ RNA, 740955, TaKaRa Bio Inc, Shiga, Japan) according to the manufacturer’s protocol. The resulting RNA was processed into cDNA using the PrimeScript^®^ RT Master Mix kit (RR036A, Takara Bio Inc.). A qRT-PCR analysis was performed using the PowerUp™ SYBR™ Green Master Mix (A25742, Applied Biosystems Japan Ltd., Tokyo, Japan) and automated sequence detection systems (StepOne, Applied Biosystems Japan Ltd.). Relative gene expression was measured using the previously validated primers for the *REST* [39], *IL-1β* [40], *TNFα* [41], *Npas4* [42], *Fos* [43], *Arc* [44], *Egr2* [45], *Nr4a1* [25], *holo-GR* [29], and *Dnmt3a* [46] genes (Table 4). Furthermore, cDNA derived from transcripts encoding β-actin was used as the internal control.

### 4.5. Statistical Analysis

Statistical analysis for cognitive activity was performed using a one-way ANOVA. Confidence intervals and significant differences in means were estimated by using Tukey’s honestly significant difference method and Fisher’s exact probability test.

## 5. Conclusions

In control group-housed older mice, increased expression of the REST and Npas4 down-regulators, holo-GR and Dnmt3, led to a repressed state of Npas4 transcription. Theanine suppressed holo-GR and Dnmt3, resulting in a higher expression of Npas4. This may have fine-tuned the excitation/inhibition balance, resulting in a reduced stress response.

## Figures and Tables

**Table 1:** The top 10 functions that were significantly down- or upregulated following theanine ingestion.

Expression	Function	Genes	Contents
Downregulated	Biological process	1623	37,720
	Regulation of transcription, DNA-templated	1609	18,613
	Positive regulation of transcription from RNA polymerase II promoter	650	9631
	Transcription, DNA-templated	587	12,890
	Signal transduction	473	12,057
	Transport	465	12,634
	Positive regulation of transcription, DNA-templated	387	6212
	Cell adhesion	384	3802
	Metabolic process	377	10,709
	Multicellular organismal development	344	6594
Upregulated	Regulation of transcription, DNA-templated	647	18,613
	Transcription, DNA-templated	527	12,890
	Positive regulation of transcription from RNA polymerase II promoter	503	9631
	Translation	465	2523
	Transport	352	12,634
	Metabolic process	335	10,709
	Negative regulation of transcription from RNA polymerase II promoter	234	6790
	Protein phosphorylation	232	8086
	Multicellular organismal development	229	6594
	Phosphorylation	219	5556

**Table 2:** The top 10 genes that were significantly down- or upregulated following theanine ingestion.

Expression	Symbol	Full Name
Downregulated	Ttr	transthyretin
	Kcnj13	potassium inwardly rectifying channel, subfamily J, member 13
	Npas4	neuronal PAS domain protein 4
	Fos	FBJ osteosarcoma oncogene
	Arc	activity regulated cytoskeletal-associated protein
	Egr2	early growth response 2
	Dusp1	dual specificity phosphatase 1
	Nr4a1	nuclear receptor subfamily 4, group A, member 1
	Gh	growth hormone
	Olfr382	olfactory receptor 382
Upregulated	Mela	melanoma antigen
	Zfp125	zinc finger protein 125
	Hbb-b2	hemoglobin, beta adult minor chain
	C1qc	complement component 1, q subcomponent, C chain
	Ly6a	lymphocyte antigen 6 complex, locus A
	Hba-a2	hemoglobin alpha, adult chain 2
	Tpm3-rs7	tropomyosin 3, related sequence 7
	Gm8615	glucosamine-6-phosphate deaminase 1 pseudogene
	Txnip	thioredoxin interacting protein
	Edv	endogenous sequence related to the Duplan murine retrovirus

**Table 3:** Experimental groups.

Housing Condition	1 M (2 Months Old)	6 M (7 Months Old)
Group	Control (water)	Control (water)
	Theanine	Theanine
Alone	Control (water)	Control (water)
	Theanine	Theanine
Two	Control (water)	Control (water)
	Theanine	Theanine

**Table 4:** Sequence of the primers used in qRT-PCR.

Gene	Forward Sequence (5′to3)	Reverse Sequence (5′to3)	Ref.
*β-actin*	TGACAGGATGCAGAAGGAGA	GCTGGAAGGTGGACAGTGAG	
*REST*	ATCGGACGCGGGTAGCGAG	GGCTGCCAGTTCAGCTTTCG	[39]
*IL-1β*	GCAACTGTTCCTGAACTCAACT	ATCTTTTGGGGTCCGTCAACT	[40]
*TNFα*	CTGTCTACTGAACTTCGGGGTGAT	GGTCTGGGCCATAGAACTGATG	[41]
*Npas4*	AGCATTCCAGGCTCATCTGAA	GGCGAAGTAAGTCTTGGTAGGATT	[42]
*Fos*	AAGTAGTGCAGCCCGGAGTA	CCAGTCAAGAGCATCAGCAA	[43]
*Arc*	ACGATCTGGCTTCCTCATTCTGCT	AGGTTCCCTCAGCATCTCTGCTTT	[44]
*Egr2*	CTACCCGGTGGAAGACCTC	AATGTTGATCATGCCATCTCC	[45]
*Nr4a1*	CTGCCTTCCTGGAACTCTTCA	CGGGTTTAGATCGGTATGCC	[25]
*holo-GR*	GATGGGGAATGACTTGGGCT	TTGGGATTCTCTGGACGGCT	[29]
*Dnmt3a*	CTGGTGATTGGAGGCAGTCCATGCA	TAGCTGAGGCTGTCTGCATCGGACA	[46]

## Data Availability

Not applicable.
